# Manipulation of habitat isolation and area implicates deterministic factors and limited neutrality in community assembly

**DOI:** 10.1002/ece3.3126

**Published:** 2017-06-20

**Authors:** Terry J. Ord, Jack Emblen, Mattias Hagman, Ryan Shofner, Sara Unruh

**Affiliations:** ^1^ Evolution and Ecology Research Centre and the School of Biological Earth and Environmental Sciences University of New South Wales Kensington NSW Australia

**Keywords:** edge effect, field experiment, island biogeography, neutral theory, niche theory

## Abstract

Theory predicts deterministic and stochastic factors will contribute to community assembly in different ways: Environmental filters should regulate those species that establish in a particular area resulting in the ecological requirements of species being the primary driver of species distributions, while chance and dispersal limitation should dictate the likelihood of species reaching certain areas with the ecology of species being largely neutral. These factors are specifically relevant for understanding how the area and isolation of different habitats or islands interact to affect community composition. Our review of the literature found few experimental studies have examined the interactive effect of habitat area and isolation on community assembly, and the results of those experiments have been mixed. We manipulated the area and isolation of rock “islands” created de novo in a grassland matrix to experimentally test how deterministic and stochastic factors shape colonizing animal communities. Over 64 weeks, the experiment revealed the primacy of deterministic factors in community assembly, with habitat islands of the same size exhibiting remarkable consistency in community composition and diversity, irrespective of isolation. Nevertheless, tangible differences still existed in abundance inequality among taxa: Large, near islands had consistently higher numbers of common taxa compared to all other island types. Dispersal limitation is often assumed to be negligible at small spatial scales, but our data shows this not to be the case. Furthermore, the dispersal limitation of a subset of species has potentially complex flow‐on effects for dictating the type of deterministic factors affecting other colonizing species.

## 1 INTRODUCTION

Our ability to perform experimental manipulations is a key challenge for studying the process and consequence of colonization in the formation of species communities in nature. Without such manipulations, it is difficult to identify the relative effects of different factors or trace shifts in community composition through time that can provide a useful perspective on the deterministic and stochastic factors that might influence local communities (e.g. environmental filtering or the role of chance in colonization; Chase 2010). Field experiments using micro/mesocosms have the potential to offer insights into the factors that influence the species composition of spatially segregated habitats. These experiments are often difficult to perform, but when possible, most researchers have sought to manipulate one of two key variables that have frequently been implicated by habitat fragmentation experiments (e.g. Haddad et al., 2015) and empirical studies of island biota (e.g. Lomolino, 1982): the effect of area and isolation of habitat “islands” on species communities (Table 1).

**Table 1:** Past experimental tests of area and isolation effects on species diversity or community composition by date of publication and variable(s) examined. Studies were identified through a systematic search of ISI Web of Science and the overlap of two searches using the topic terms of (i) “island/habitat area”, “island/habitat size”, “island/habitat isolation”, “island/habitat distance”, or “habitat fragment*” and (ii) “species richness”, “species diversity”, “species composition”, “community composition”, “community structure”, “community assembl*” or “community divers*”. The outcome of these searches was further refined by the topic term “experiment*” and restricted to the research domain of “Science Technology”. The titles and abstracts of the 7,624 articles identified were assessed manually and those found to be relevant were downloaded through the UNSW library gateway. This resulted in the detailed review of 139 papers. The 23 experiments listed (and a meta‐analysis of a subset of experiments; Haddad et al., 2015) represent those incorporating some form of manipulation relevant for documenting the effects of area and isolation. Fragmentation experiments were included if direct comparisons were made among patches of different area or isolation (not including habitat corridors)

Study	Variable (direction of effect)	Ecosystem type	Taxonomic group	Spatial scale	Experiment length (sampling frequency)	Broad objective
Simberloff (1976)	Area (+^a^)	Mangrove islands	Arthropods	Natural mangrove islands, with experimental reduction of area within the general range of 264–1,263 m^2^	3 years (annual)	Area effects on species richness, immigration and extinction, while controlling for overall habitat diversity
Schoener and Schoener (1983)	Area (+)	Oceanic islands	Lizards	Natural islands varying in area from 26 to 8,060 m^2^	5 years (5–6 months, then annually)	Area and propagule size effects on establishment success
Have (1987)	Area (+^a^)	Freshwater	Ciliates	Plexiglass cylinders with areas of 5.3 cm^2^, 10.18 cm^2^ or 21.23 cm^2^	53 days (every 2–12 days)	Area effect on species richness
Schoener and Spiller (1995)	Area (+)	Oceanic islands	Spiders	Natural islands varying in area from 11–51 m^2^ (“small”) to 167–3726 m^2^ (“large”) with spiders experimentally introduced with or without the secondary introduction of a lizard predator	12 years (4 days, 4 months, then annually)	Area and predator effects on spider abundance
Davies and Margules (1998), Davies, Melbourne, and Margules (2001)	Area (none^b^)	Eucalyptus forest	Beetles	Continuous eucalyptus forest fragmented to areas of 0.25, 0.875 and 3.062 ha with cleared matrix planted with commercial pine	4–5 years (four times annually)	Fragmentation effects on beetle diversity and abundance
Golden and Crist (1999, 2000)	Area (+^b^)	Grassland	Plants, arthropods	Grass field fragmented to patches of 1, 4, 9 and 169 m^2^	4 months (at 2 and 4 months)	Fragmentation effects on species diversity and abundance
Zschokke et al. (2000)	Area (+^b^)	Grassland	Plants, arthropods	Grass field fragmented to patches of 0.25 m^2^, 3 m^2^ or 9 m^2^	3 years (three periods in final year)	Patch area and fragmentation effects on species diversity and abundance
Parker and Mac Nally (2002)	Area (none)	Grassland	Arthropods	Starting habitat of 225 m^2^ reduced in continuous or fragmented area	3 months (every 2–4 weeks)	Effect of habitat loss and fragmentation on species richness and abundance
Jelbart et al. (2006)	Area (variable)	Marine	Fish	Natural seagrass beds of varying size from 2,290 to 211,170 m^2^ and an experiment using artificial beds of 7.2 m^2^ or 13 m^2^ with an independent manipulation of edge‐to‐interior ratio	Observational: <1 year (once in autumn and once in spring); experimental beds: 39 days (once at conclusion of experiment)	Area and edge effects on species richness and abundance
With and Pavuk (2011, 2012)	Area (+)	Agriculture field (red clover monoculture)	Arthropods	Fields initially 256 m^2^ reduced by 20%–90% in continuous or fragmented area	3 years (2–3 times annually)	Area and fragmentation effect on arthropod diversity
Montana, Layman, and Winemiller (2015)	Area (+)	Freshwater	Fish, aquatic invertebrates	Artificial habitats constructed on flood plain sand banks using tiles or stacked hollowed bricks ranging in total area of 160–10,730 cm^2^	21 days (once at conclusion of experiment)	Area effects on species diversity in a dynamic flood environment
Simberloff and Wilson (1969, 1970)	Isolation (−a)	Mangrove islands	Arthropods	Natural mangrove islands that ranged in distance from 2 to 533 m from mainland	360 days (every 20–80 days)	Colonization dynamics of returning arthropod communities following fumigation
Arrington, Winemiller, and Layman (2005)	Isolation (variable)	Freshwater	Fish, aquatic invertebrates	Artificial habitat patches constructed on flood plain sand banks from hollowed bricks positioned at 25, 75 and 225 m from an aquatic source habitat, with an additional experimental manipulation of patch complexity (brick holes left open or plugged)	21 days (once at conclusion of experiment)	Effect of habitat isolation and habitat complexity on species diversity in a dynamic flood environment
Chase et al. (2010)	Isolation (variable)	Freshwater	Phytoplankton, zooplankton, arthropods, gastropods, tadpoles	Equal sized mesocosms (1,130 L stock tanks) positioned at 5 m or 200 m from a natural water body, with experimental introductions of predatory fish to half the mesocosms	2 years (annually)	Isolation effect on trophic cascades
Hein and Gillooly (2011; see also Fahimipour & Hein, 2014)	Isolation (−)	Freshwater mesocosms	Arthropods	Artificial ponds positioned at 10, 100 or 400 m from natural lakes	8 weeks (every 2 weeks)	Effect of isolation on colonization rates of trophic guilds (prey vs. predator taxa), and a later examination of food web assembly (Fahimipour & Hein, 2014)
Astrom and Part (2013)	Isolation (variable)	Moss microcosms	Arthropods	Equal sized microcosms consisting of moss habitat islands (100 cm^2^) placed on a gravel or plywood landscape with half connected by corridors	103 days (once at study conclusion)	Impact of habitat corridors, environment of surrounding matrix and environmental disturbance (defaunated and subsequent freezing) on taxon abundance and diversity
Fahimipour and Anderson (2015)	Isolation (−)	Freshwater	Arthropods, zooplankton	Equal sized mesocosms (wading pools) positioned at 30 or 300 m from a natural lake with experimental introductions of predatory fish to half the mesocosms	12 weeks (every 2 weeks)	Isolation effects on trophic cascades in the presence/absence of a predator
Holt, Robinson, and Gaines (1995); Cook, Lane, Foster, and Holt (2002); Cook, Yao, Foster, Holt, and Patrick (2005)	Area (variable), isolation (−)	Grassland	Plants	Patch areas of 32 m^2^, 288 m^2^ or 0.5 ha positioned either near (<150 m) or far (>150 m) from remnant forest	Up to 18 years (1–3 times annually)	Effect of fragmentation on plant succession
Spencer and Warren (1996)	Area (+), isolation (−)	Freshwater	Bacteria, Protista	Laboratory microcosms consisting of petri dishes of either 17 cm^2^ or 143 cm^2^ in size with isolation varied by experimental introduction (yes or no), and an additional manipulation of the nutrient environment (high or low)	46 days (at conclusion of experiment)	Area, immigration and environment quality effects on food webs
Lonzarich, Warren, and Lonzarich (1998)	Area (none), isolation (none)	Freshwater	Fish	River pools separated by <10 m or >10 m from an adjacent source pool, with pools naturally varying in area	40 days (1 day, 3 days, and then every 10 days)	Recovery of fish communities following experimental removal
Laurance et al. (2002, 2011; reviewed by)	Area (+), isolation (variable)	Forest	Plants, arthropods, amphibians, birds and mammals	Forest fragmented by farmland clearing into patches of 1, 10 and 100 ha at distances of 80–650 m from remnant continuous forest	Up to 32 years (various)	Effect of fragmentation on various aspects of a forest community
Kotiaho and Sulkava (2007)	Area (+), isolation (−)	Humus microcosms	Nematodes	Humus habitat islands of 95 or 855 mm^2^ placed in an artificial sand matrix at 10 or 30 mm from undisturbed forest floor, with experimental introductions of predatory mites to half the microcosms	62 days (at conclusion of experiment)	Area, isolation and predator effects on colonization
Harvey and MacDougall (2014, 2015)	Area (+), isolation (variable)	Grassland	Plants, arthropods	Grass patches of 25, 100 and 400 m^2^ positioned from 8 to 330 m from a remnant grass field, with additional manipulations of soil nutrient level and mechanical disturbance	6 months (2014; plants once at ~4 months, arthropods at ~5 months and ~6 months) and ~2 years (2015; once at study conclusion)	Area and isolation effects on trophic guilds (2014), and their interaction with environmental quality and disturbance (2015)
Haddad et al. (2015; meta‐analysis by)	Area (+), isolation (−)	Terrestrial, various	Plant, insect, animal	Habitat patches at various spatial scales, from cm to hectares	Years to decades (various)	Meta‐analysis of seven long‐term fragmentation experiments

a No formal statistical test performed; interpretation based on observed trends.

b Sampling effort not standardized among area treatments, which may have impacted final results.

This emphasis on habitat area and isolation has deep roots in classical island biogeography theory that attempted to explain differences in species diversity among islands as a function of their size and distance from mainland sources (MacArthur & Wilson, 1967; see also recent reviews in Losos & Ricklefs, 2009). Heavily influential on much of the thinking in community ecology in the seventies and eighties (as it arguably still is today; Hubbell, 2009), the application of island biogeography theory to nonisland settings of habitat patches in mainland environments was intuitive, but controversial (reviewed by Laurance, 2009). Today, the differences between oceanic islands and isolated habitat patches are well recognized (e.g. see Haila, 2002; Laurance, 2008, 2009). Nevertheless, an enduring legacy of classical island biogeography theory continues to be the expected impact of area and isolation on species diversity, which has proven robust in a range of ecological settings (Haddad et al., 2015; Hanski, 2009; Schoener, 2009).

Our review of the experimental literature on this topic has also revealed that, while most ecological manipulations have tested differences in habitat area (most commonly) or habitat isolation (less frequently), few have explicitly tested the interaction of area and isolation on community diversity (Table 1). This is surprising for two reasons. First, classical island biogeography theory, which has so often provided the inspiration for many of these studies, emphasizes the interaction of both area and isolation on the underlying dynamics that shapes species diversity on islands (MacArthur & Wilson, 1967). Second, and more recently, the debate surrounding the relative contribution of deterministic and stochastic factors in community ecology make contrasting predictions about how species communities should differ as a function of habitat area and isolation. For example, a deterministic perspective considers the composition of localized communities as the outcome of ecological factors such as environmental filtering (niche‐based models: Leibold, 1995; Tilman, 2004; Soberon, 2007) and competition between invaders and residents (limiting similarity or niche partitioning: MacArthur & Levins, 1967; Tilman, 1997; Levine & HilleRisLambers, 2009; review by Chase & Leibold, 2003). The alternative view is that chance coupled with dispersal limitation interact to dictate the likelihood of species reaching habitats of different size and isolation (classical island biogeography: MacArthur & Wilson, 1967; and its extension by neutral theory: Hubbell, 2001; Rosindell, Hubbell, & Etienne, 2011; NB: dispersal limitation is not a specific requirement of neutrality per se, but is expected to be a key factor when comparing among habitats that differ in connectivity). That is, the presence of a species is either the product of abiotic and biotic conditions in a habitat, and unrelated to the size or isolation of that habitat (determinism), or dependent on chance dispersal to a habitat–with colonization expected to be more likely for larger and less isolated habitats–and less related to the conditions of that habitat (stochasticity/neutrality). The reality is probably somewhere between these two extremes (Chisholm, Fung, Chimalakonda & O'Dwyer, 2016; Hanski, 2009), and the focus has now shifted toward documenting the relative contribution of deterministic and stochastic effects (e.g. Ward & Thornton, 2000; Chase, 2007, 2010; Fahimipour & Anderson, 2015; Li et al., 2016; Passy, 2016).

On a basic level, the limited number of manipulations of both habitat area and isolation in the same experiment represents a gap in our general understanding of how these variables interact to influence species diversity at spatial scales that are relevant in nature (Table 1). What we do know is that habitat area generally has a positive effect on species diversity (Table 1), but this might occur because there is an increased likelihood of taxa dispersing to larger patches (e.g. see Buckley & Knedlhans, 1986; Lomolino, 1990) or because larger habitats have greater niche diversity (Ricklefs & Lovette, 1999). The influence of habitat isolation or connectivity on species diversity is more variable, with richness sometimes decreasing or not changing at all (Table 1). Theory generally predicts that reducing habitat isolation should compensate for small habitat area (reviewed by Hanski, 2009; Lomolino, Brown, & Sax, 2009), and vice versa, but this has rarely been experimentally tested. The effect of area and isolation on community composition (not simply its richness) is even less clear.

In this study, we performed a manipulative field experiment to test the interacting effects of habitat area and isolation on the species richness and composition of localized animal communities on newly created habitat “islands” positioned in a grassland environment. These islands consisted of subsoil mounds covered with bush rock and deadwood that were initially devoid of all vegetation and any obvious sign of arthropod or other animal activity. Islands were either small or large and placed either near or far from open sclerophyll forest in which rocky outcrops and deadwood from fallen branches and trees were common. Special attention was made on keeping the environments on islands consistent to ensure differences among islands were limited to variables associated with area and isolation. Colonization of these habitat islands was tracked over 64 weeks.

**Figure 1:**
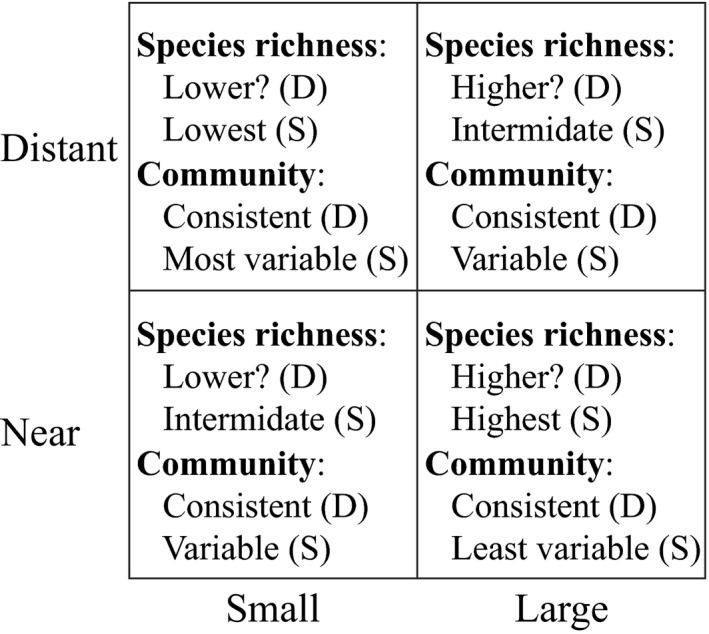
Predicted species diversity and community similarity of habitat islands of different area and isolation under deterministic (D) or stochastic (S) models of community assembly

We had several predictions on how species composition should differ among islands depending on the relative contribution of deterministic and stochastic influences on colonization (Figure 1). Given enough time for colonization to occur, the overriding effect of deterministic processes should be the accumulation of similar numbers and combinations of species on all islands, irrespective of isolation and to some extent area. This is because the environments on all of our islands were intentionally designed to be alike (i.e. possess the same range of microhabitats/niche diversity). Nevertheless, habitat edges can have complex effects on the composition of patch communities (Debinski & Holt, 2000; Golden & Crist, 2000; Jelbart, Ross, & Connolly, 2006; Laurance et al., 2011; Orrock, Curler, Danielson, & Coyle, 2011; With & Pavuk, 2012), and the ratio of edge‐to‐interior on our experimental islands was higher on small islands than large islands (by 2:1). The extent to which this might affect island communities was unclear, but at the very least species number and composition should be similar among islands of the same size (and irrespective of distance; Figure 1).

In contrast, classical island biogeography and modern neutral theory (in the context of immigration) assumes all taxa are ecologically equivalent, and the composition of communities depends primarily on chance (Chase & Myers, 2011). Larger islands should therefore have consistently higher species richness than smaller islands, and near islands should have higher species richness than distant islands. This in turn predicts the greatest diversity will be concentrated on large, near islands, while the lowest diversity should occur on small, distant islands (Figure 1). The predicted species richness on small, near islands and large, distant islands was unclear and would depend on the relative magnitude of size and isolation effects. If similar, species richness on these islands should be intermediate and roughly equivalent. In terms of community composition, our island communities should be highly variable, especially among small, isolated islands where ecological “drift” is expected to be highest (Figure 1). Conversely, communities should tend to be more similar among large, near islands because dispersal to these islands is expected to be the least restricted from the adjacent forest “mainland” (Figure 1). It was also possible that temporal convergence in the combination of species occurring on habitat islands might start to occur, especially among those of the same type, given the likelihood that taxa will eventually find themselves on even the most distant island should increase with time. That is, dissimilarity among communities on islands of the same size and isolation should decrease over time. This temporal shift should be most noticeable on large, near islands and least on small, distant islands (whereas under a deterministic model, any temporal shifts in the combination of species should be consistent among all habitat islands; e.g., because of seasonal changes in community composition). Finally, even in the absence of dispersal limitation, a purely neutral assemblage of species should result in little similarity among any of the islands.

## 2 MATERIALS AND METHODS

### 2.1 Experimental design

The experiment was conducted on a private property near the locality of Wollar in the central tablelands of New South Wales, Australia. The property had large areas of cleared pasture that had been used for low‐density cattle farming for several decades up until early 2007. This grassland environment transitioned abruptly into remnant dry sclerophyll eucalyptus forest with an open understory scattered with rock outcrops and deadwood from fallen trees and branches.

**Figure 2:**
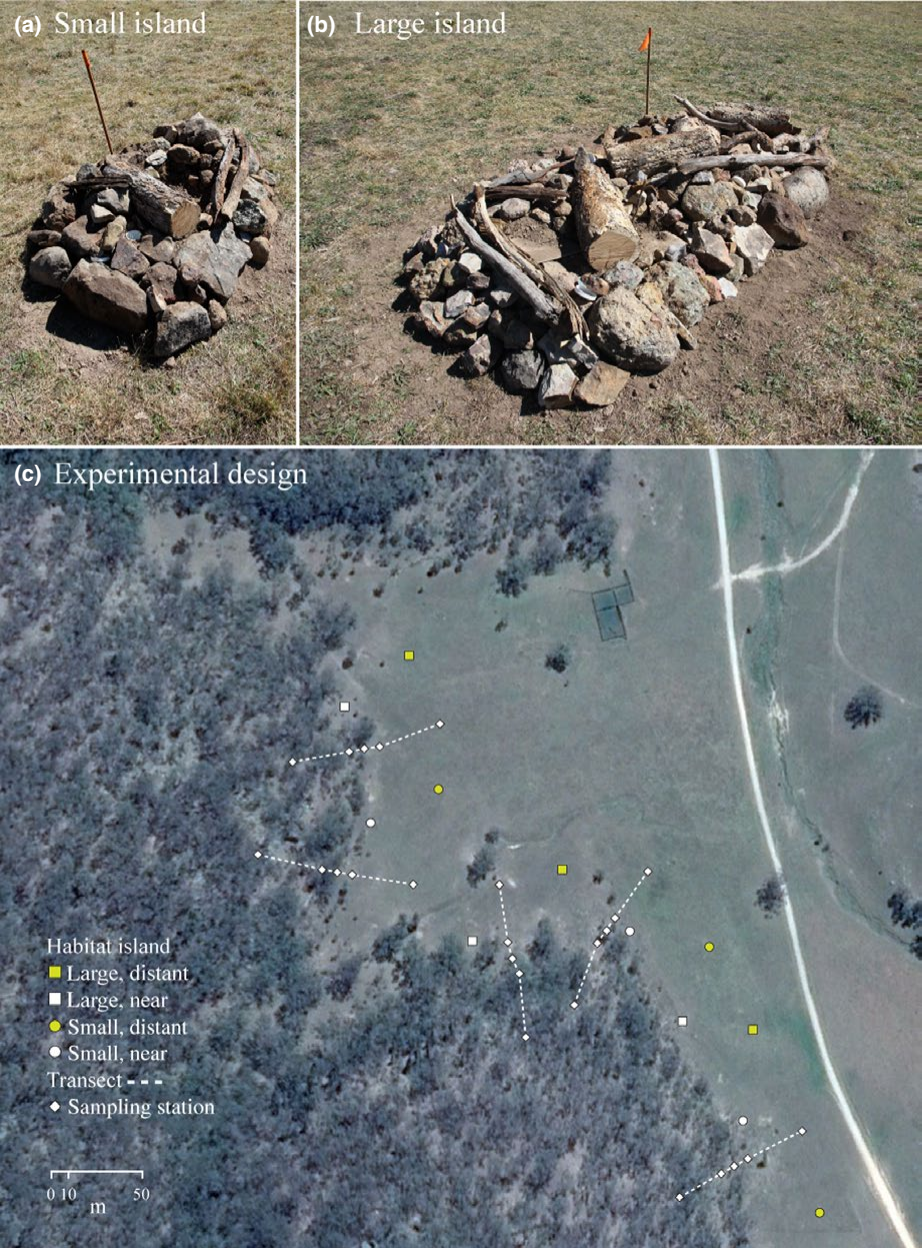
Experimental habitat islands (a) small (1.08 m^2^) and (b) large (4.32 m^2^) and their (c) positions in the grassland matrix relative to adjacent sclerophyll forest. Also shown are the positions of transects used to quantify animal communities in both forest and grassland environments

Two habitat island sizes were constructed in the grassland environment in September 2013 by placing a thin line of sand to outline a rectangle of 0.6 by 1.8 m (1.08 m^2^; small) or 1.2 × 3.6 m (4.32 m^2^; large; Figure 2a,b). The longest edge of the island was angled parallel to the forest boundary and a TruPulse 200 laser range finder used to position the edge of the island to a distance of either 10 m (near) or 50 m (distant) from the forest drip‐line (Figure 2c). All islands were separated from one another by a distance of >75 m so the closest source of potential colonizers was from the eucalyptus forest “mainland”. An excavator was then used to pile subsoil to a maximum height of approximately 0.3 m (small islands) or 0.6 m (large islands), which was then manually covered in a layer of bush rock. Deadwood was then placed systematically onto the island with the amount dependent on the size of the island: either one or four large pieces of tree trunk chain‐sawed into approximately 1‐m lengths and a combination of large and small branches (Figure 2a,b). Subsoil and bush‐rock were sourced from the grassland matrix, as was the deadwood that was taken from a standing dead eucalyptus tree approximately 20 m from the forest boundary. Three replicate islands were constructed for each size and distance treatment, for a total of 12 habitat islands (Figure 2c).

Habitat islands were surveyed by pooling data from three sampling methods: (1) large “dry” pitfalls with an opening diameter of 25 cm sunk to a depth of 60 cm; (2) small “wet” pitfalls with an opening diameter of 10 cm filled with 100–200 ml of water; and (3) fly‐paper glue‐traps laid flat onto the substrate of the island. To ensure consistent sampling effort across island sizes, one or four replicates for each method were used on small or large islands, respectively. Five permanent transects were also established at the time islands were constructed, with survey stations placed at −50, −10, 0, 10, and 50 m relative to the forest–grassland boundary (Figure 2c). Each station consisted of one dry pitfall, one wet pitfall and one fly‐paper glue trap laid flat to the ground. All pitfalls were permanent and embedded with the opening flush to the ground during the initial construction of habitat islands and transects. During survey periods, pitfalls were left open for four days and cleared daily. Taxa found in dry pitfalls (large centipedes, spiders, lizards, snakes, and frogs) were noted and photographed for identification and released back onto habitat islands or immediately adjacent to the transect station. Taxa collected in wet pitfalls (primarily terrestrial arthropods) were transferred to specimen jars filled with 80% ethanol. All pitfalls were kept sealed outside of survey periods. Fly‐paper glue‐traps (collecting primarily flying arthropods) were only deployed during survey periods and left for two days before being collected and stored in a freezer until specimen identification. Comprehensive sampling using all three methods was conducted at 5, 12, 19, and 28 weeks post island construction, while the final survey period at 64 weeks used only dry and wet pitfalls.

Specimens collected using wet pitfalls and fly‐paper glue‐traps were sorted into morpho‐species and individuals counted with the aid of a dissecting microscope. Photographs of specimens trapped in dry pitfalls were used to identify taxa to morpho‐species or occasionally to genera or species for reptiles. Because specimens from dry pitfalls were returned to islands or the matrix surrounding a transect station and not individually marked before release, we used the maximum number of individuals trapped in 1 day as our measure of abundance for a given taxon for a given survey period.

### 2.2 Statistical analyzes

We used the “vegan” package ver 2.3‐4 (Oksanen et al., 2016) implemented in R ver 3.2.4 (R Development Core Team, R Foundation for Statistical Computing, Vienna) to compute three diversity indices: inverse Simpson dominance, Shannon‐Weaver diversity and total number of morpho‐species. Our sampling protocol was not designed to be exhaustive rather to give a consistent and representative snapshot of taxa occupying habitat islands over several days during each survey period. This was expected to provide a reasonable picture of the diversity of common taxa, but potentially limited in the detection of rare taxa. In this sense, Simpson dominance should provide the most robust estimate of species richness for our experimental design because it is the least sensitive to reliably detecting rare taxa (see Lande, DeVries, & Walla, 2000). The Shannon index is slightly more sensitive to the presence of rare taxa, while the total number of morpho‐species is the most sensitive to the accurate detection of rare taxa. Regardless, the main objective of comparing results across all three indices was to provide a general view of how taxon diversity as a function of abundance equality differed among islands.

To analyze these differences, diversity indices were entered into a log‐likelihood linear mixed‐effects model in the R package “lme4” ver 1.1‐8 (Bates, Maechler, Bolker, & Walker, 2015). This model included fixed effects for island size (0, small; 1, large), isolation (0, near; 1, distant) and their interaction, and a random intercept and slope for sampling period (week 5, 12, 19 and 28). Data from week 64 were analyzed separately in a standard fixed effects linear model because it only included pitfall data.

The composition of morpho‐species communities was visualized using nonmetric multidimensional scaling (NMDS) based on Bray–Curtis dissimilarity. This was implemented with the “metaMDS” wrapper function in the “vegan” package. The position of each habitat island was then presented in an ordination plot with replicates joined by convex hulls. Weeks 5, 12, 19, and 28 were evaluated collectively in the same ordination, while week 64 was subject to an independent analysis and presented separately.

Statistical comparisons were also made of Bray–Curtis dissimilarities using a multivariate permutation ANOVA implemented with the “adonis” function in the “vegan” package. Tests were based on 999 permutations and included fixed effects for island size, isolation, survey period week, and their interactions (NB: a mixed‐effects model comparable to those applied to diversity indices that included week as a random effect was not possible in this model's structure). The order of fixed effects entered into the model was varied to examine the sensitivity of the model to the sequence of entered variables but was found not to change the interpretation of results (i.e., results were qualitatively unchanged). We also compared island communities to the surrounding grassland matrix using permutation tests of the dissimilarity of small and large islands relative to transect stations at comparable distances from the forest boundary. In these tests, Bray–Curtis dissimilarities were computed based on proportional abundance of morpho‐species rather than absolute abundance to compensate for differences in sampling effort between islands and the grassland matrix. Fixed effects included habitat (0, matrix; 1, island), distance from forest boundary (0, near; 1, distant), and week of sampling (5, 12, 19 and 28). Permutation tests comparing communities among islands, or between islands and the grassland matrix, were conducted separately for data collected in week 64.

Finally, we applied the “betadisper” function in “vegan” based on 999 permutations to examine differences in Bray–Curtis dissimilarity among islands as a function of treatment for each survey period (treatment was specified as “large, distant,” “large, near,” “small, distant,” and “small, near”). More specifically, this analysis provided a means of testing the prediction that large, near islands were more similar in composition than small, distant islands, and how this similarity might have changed over time (see the conclusion of Section 1 and Figure 1).

## 3 RESULTS

### 3.1 Species richness

**Figure 3:**
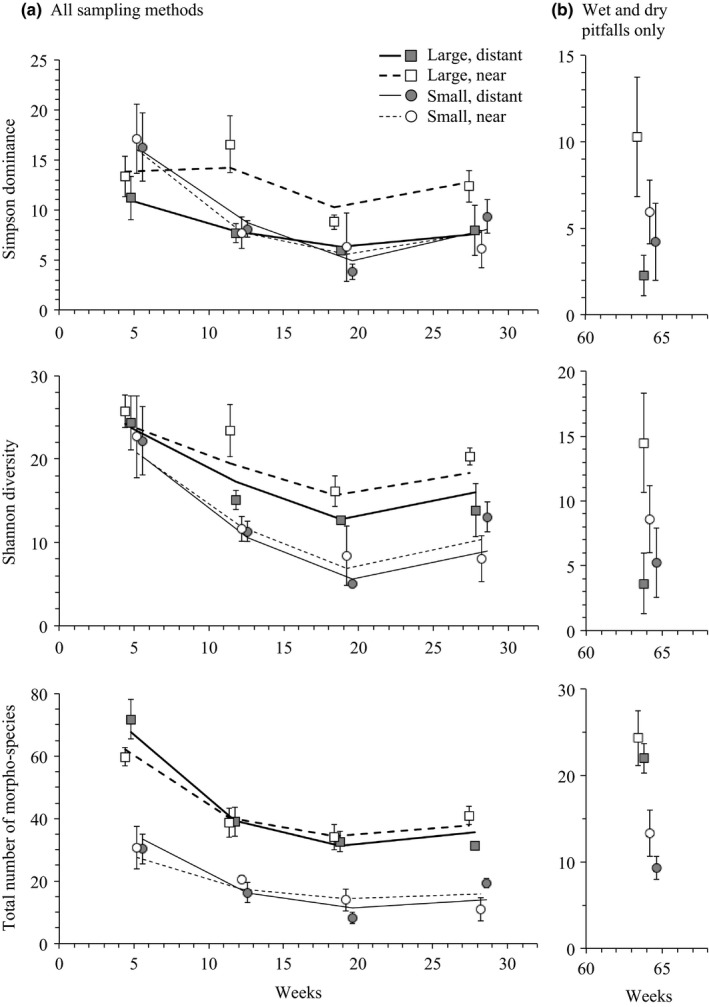
Changes in the diversity of animal communities on habitat islands (a) within the first 28 weeks based on all sampling methods and (b) in the final survey period of week 64 that only used data from pitfall traps. Data shown are means with standard errors of three replicate islands. Lines depict computed trends from mixed‐effect models reported in Table 2

**Figure 4:**
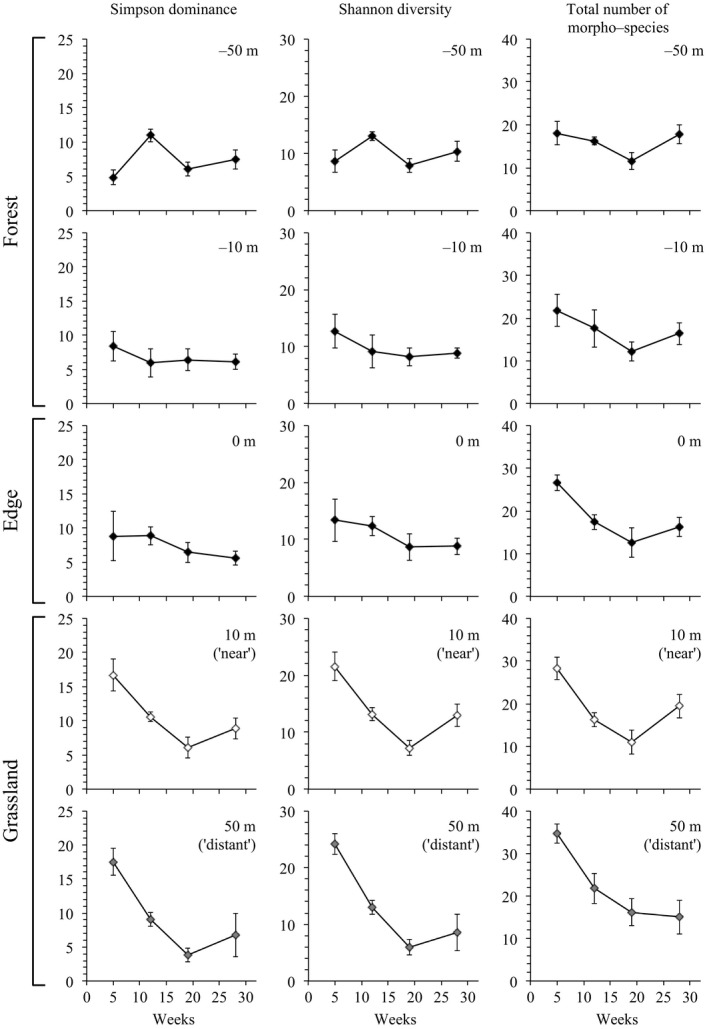
Changes in the diversity of animal communities along transects positioned perpendicular to the forest–grassland boundary. Sampling stations were positioned at five points (Fig. 2): inside the forest at 50 and 10 m, at the forest–grassland boundary at 0 m, and out in the grassland matrix at 10 and 50 m. The latter positions corresponded with distances of habitat islands near and distant, respectively. Data shown are means with standard errors across five replicate transects

The initial influx of animal taxa to habitat islands evidently occurred before the first survey period in week 5, after which taxon numbers on most islands decreased before tending to stabilize in later stages of the experiment (Figure 3; NB: vegetation was initially absent on islands but increased steadily over the course of the experiment; Fig. S1). A dip in richness across survey periods was also apparent in the surrounding grassland matrix and consistent with a general seasonal effect on animal communities in the grassland environment as a whole (Figure 4).

Overall, habitat island size generally had the greatest effect on diversity when measured with absolute numbers of morpho‐species or Shannon diversity (Tables 2b,c and 3c; Figure 3). However, Simpson dominance suggested a strong interaction between area and isolation (Table 2a) with the highest diversity of common taxa occurring on large, near islands (Figure 3a). This effect was consistent for most survey periods after week 5 (Figure 3). Results from week 68 that only included data from pitfall surveys suggested a negative effect of isolation on species number over island size for Simpsons and Shannon estimates (Table 3a,b; Figure 3b).

**Table 2:** Mixed‐effect models of diversity as a function of habitat island area and isolation based on all sampling methods in weeks 5–24. Diversity was measured as (a) Simpson dominance, (b), Shannon‐Weaver diversity or (c) total number of morpho‐species. Variables with 95% confidence intervals (CIs) that do not overlap zero are highlighted in bold. An interaction of area and isolation was initially considered in all models but removed if not demonstrating a statistically distinguishable effect

(a) Simpson dominance	
Random effects (variance among weeks)	
Variable	Effect size (*z*)
Intercept	4.22
Island area	3.87
Island isolation	0.85
Island area × isolation	2.57
*Residual*	3.39

Fixed effects		
Variable	Estimate (lower 95% CI, upper 95% CI)	Effect size (*t*)
Intercept	9.30 (4.74, 13.85)	4.00
Island area	3.47 (−1.19, 8.12)	1.46
Island isolation	0.08 (−2.76, 2.91)	0.05
**Island area** × **isolation**	−**4.65 (**−**9.24,** −**0.07)**	−**1.99**

(b) Shannon diversity	
Random effects (variance among weeks)	
Variable	Effect size (*z*)
Intercept	0.41
Island area	0.25
Island isolation	0.07
*Residual*	0.31

Fixed effects		
Variable	Estimate (lower 95% CI, upper 95% CI)	Effect size (*t*)
Intercept	2.44 (2.02, 2.87)	11.19
**Island area**	**0.51 (0.21, 0.81)**	**3.34**
Island isolation	−0.12 (−0.31, 0.07)	−1.24

(c) Morpho‐species number	
Random effects (variance among weeks)	
Variable	Effect size (*z*)
Intercept	5.30
Island area	5.72
Island isolation	3.41
*Residual*	6.56

Fixed effects		
Variable	Estimate (lower 95% CI, upper 95% CI)	Effect size (*t*)
Intercept	18.88 (12.77, 24.98)	6.06
**Island area**	**24.67 (17.95, 31.39)**	**7.19**
Island isolation	−0.08 (−5.08, 4.91)	−0.03

**Table 3:** Fixed‐effect models of diversity as a function of habitat island area and isolation based on pitfall data in week 64. Diversity was measured as (a) Simpson dominance, (b), Shannon‐Weaver diversity or (c) total number of morpho‐species recorded. Variables with large statistically effects are highlighted in bold. An interaction of area and isolation was initially considered in all models but removed if not demonstrating a statistically distinguishable effect

Variable	Estimate	Effect size (*t*)	*p*
(a) Simpson dominance: *F*_2,9_ = 2.11, adjusted *r* ^2^ = .17, *p *=* *.18			
Intercept	7.49	3.56	0.006
Island area	1.20	0.49	0.63
Island isolation	−4.85	−1.99	0.08
(b) Shannon diversity: *F*_2,9_ = 2.95, adjusted *r* ^2^ = .26, *p *=* *.10			
Intercept	2..28	5.33	0.0005
Island area	0.06	0.12	0.90
** Island isolation**	−**1.20**	−**2.42**	**0.04**
(c) Morpho‐species number: *F*_2,9_ = 15.11, adjusted *r* ^2^ = .72, *p *=* *.001			
Intercept	12.92	6.69	<0.0001
** Island area**	**11.83**	**5.31**	**0.0005**
Island isolation	−3.17	−1.42	0.19

### 3.2 Community composition

There was limited overlap between communities surveyed on islands to those found in the surrounding grassland matrix (Table S1). Approximately 70%–80% of the communities recorded on islands were distinct from the grassland community (Table S1, Fig. S2).

**Figure 5:**
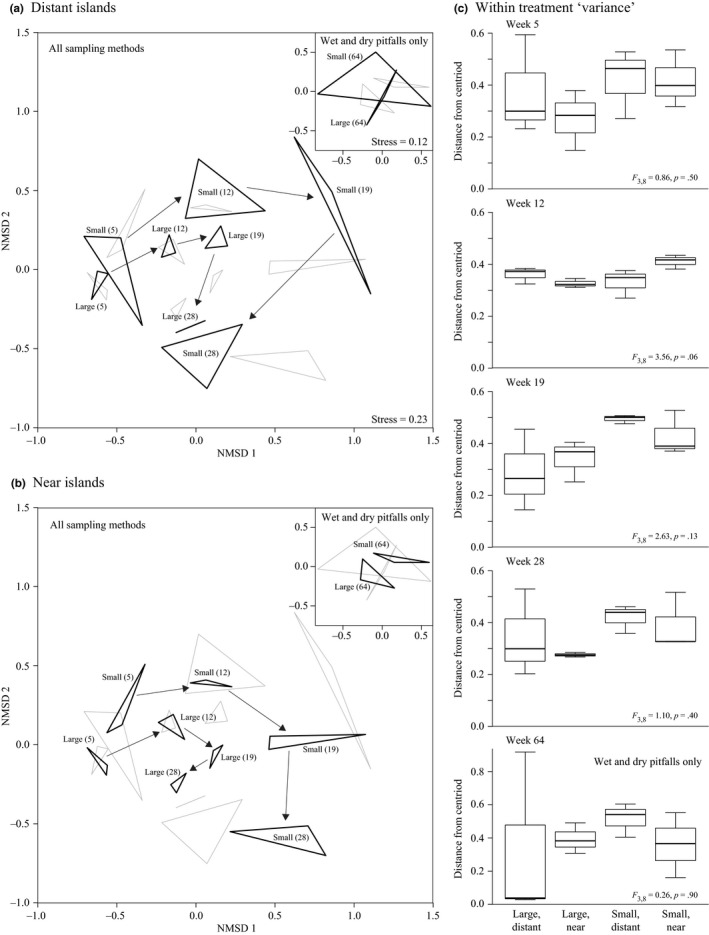
Community dissimilarity among habitat islands. Nonmetric multidimensional scaling (NMDS) plots emphasize community differences among large and small islands positioned in the grassland matrix either (a) distant or (b) near the adjacent forest habitat. Boxplots (c) show the degree of dissimilarity among island replicates within treatments and corresponding results of permutation ANOVAs

On habitat islands, community composition progressively shifted over time in ordination plots (Figure 5a,b), and this was confirmed by a large statistical effect for survey week in permutation analyzes (*r* ^2^ = .32; Table 4a). Although communities on large, near islands seemed to be more similar than small, distant islands on most occasions, there was no statistical distinguishable effect of treatment in any survey period (Figure 5c). There was also no obvious indication of a convergence in community similarity over time, either across or within particular treatments (Figure 5c).

**Table 4:** Permutation ANOVAs of community dissimilarity as a function of habitat island area, isolation, and week of survey. Data in weeks 5–24 (a) used all sampling methods, while data in week 64 (b) was based on pitfall traps only. Variables with large statistical effects are highlighted in bold

Variable	*df*	*F*	Effect size (*r*)	*p*
(a) All sampling methods, weeks 5–28				
** Week**	**1**	**5.99**	**.32**	**.001**
** Island isolation**	**1**	**1.6**	**.16**	**.04**
** Island area**	**1**	**5.97**	**.32**	**.001**
Week × island isolation	1	1.02	.13	.42
** Week** × **island area**	**1**	**1.87**	**.18**	**.02**
Island isolation × area	1	1.27	.15	.17
Week × island isolation × area	1	1.39	.15	.09
*Residual*	40			
*Total*	47			
(b) Wet and dry pitfalls, week 64				
Island isolation	1	1.31	.33	.22
Island area	1	2.09	.41	.08
Island isolation × area	1	0.93	.28	.45
*Residual*	8			
*Total*	11			

Overall, islands generally exhibited the highest similarity in community composition with other islands of the same area (*r* ^2^ = .32–.41) and, to a lesser extent, isolation (see below). Large islands generally occupied adjacent positions in ordination plots and mostly irrespective of isolation (Figure 5a,b). Small islands also tended to cluster together but were generally more variably distributed in ordination plots and tended to exhibit greater temporal shifts from one survey period to the next than large islands (Figure 5a,b; this was consistent with a prominent week by island area interaction–see Table 4a). Island isolation was also computed to have a moderate statistical effect on community composition for most survey periods (*r* ^2^ = .16 in weeks 5–28), but the direction of this effect was unclear from ordination plots.

## 4 DISCUSSION

The outcome of our experiment was broadly consistent with predictions from both deterministic (e.g. niche‐based/environmental filtering) and stochastic (island biogeography/neutral) models of community formation (Figure 1), but deterministic factors clearly dominated our results. The overriding effect of habitat area in most of our analyzes–in which islands of the same area were found to have similar estimates of taxon richness (Figure 3) and community composition (Figure 4)–was predicted if deterministic influences, and habitat edge effects in particular, were influential in shaping animal communities (Figure 1). Small habitat islands had a higher ratio of edge‐to‐interior than large islands (2:1), and fragmentation studies have reported strong effects of increased habitat edge on the colonization and species composition of habitat patches (Debinski & Holt, 2000; Laurance et al., 2002). This also appears to have been the case in our experiment.

There were also signs of environmental filtering in the temporal shifts in community composition on all islands over the course of the experiment. While island biogeography theory predicts communities will exhibit stochastic turnover of species through time (reviewed by Schoener, 2009), the changes documented in our experiment were typical of seasonal shifts in animal communities in the grassland ecosystem more broadly (Figure 4; such seasonal fluctuations in arthropod diversity in Australian grasslands are not unusual: e.g. see Parker & Mac Nally, 2002). This was despite the composition of island communities being largely distinct from that of the surrounding matrix (Fig. S2), which implicates overarching fluctuations in environmental conditions are almost certainly responsible for the changes in animal communities on both islands and the surrounding matrix. This was further supported by the consistency of community changes among island replicates within treatments (Figure 5a,b), and the lack of evidence that islands of a particular area or isolation became progressively less variable in community composition over time (which was predicted if stochastic factors were influential; Figure 5c).

Nevertheless, evidence that dispersal limitation had some influence on the composition of our habitat island communities was apparent from the interaction of area and isolation on estimates of Simpson dominance (Table 2a) and, to some extent, the tendency for isolation to be negatively associated with diversity indices more generally (e.g. Tables 2b and 3a,b). The highest number of common taxa was recorded on large, near islands (Figure 3), whereas dispersal limitation appeared to have led to the reduced number of common taxa occurring on other islands. Comparison among estimates of Simpson, Shannon and morpho‐species number helps clarify the underlying colonization dynamics that resulted in this difference. Although large habitat islands in general had a similar combination of morpho‐species (Table 4; Figure 5a,b), and almost double the number occurring on small islands (Figure 3, lowest panel), the local abundance of those morpho‐species was affected by isolation. The bulk of individuals reaching large, distant islands were limited to a subset of morpho‐species, and to such an extent that the number of dominant taxa on large, distant islands dropped to numbers more typical of those found on small islands (Figure 3, top panel). That is, chance and dispersal limitation resulted in higher abundance inequality on hard to reach habitat islands–a skewed distribution of individuals among taxa–rather than dictated which taxa were present on islands more generally. This interaction of habitat area and isolation was therefore only evident in diversity measures that accounted for differences in local abundance of taxa (Simpson dominance).

Abundance inequalities can also occur through environmental filtering. Communities in less favorable environments–for example, areas of low productivity (Chase 2010, Passy, 2016) or subject to periodic environmental stressors (Chase, 2007; Kneitel & Chase, 2004)–are subject to stronger environmental filtering. The result can be the increasing dominance of a handful of tolerant species as conditions deteriorate (Chase, 2007; Kneitel & Chase, 2004; Passy, 2016). In contrast, communities found in more favorable environments are more likely to have species compositions that reflect stochastic processes in colonization history (Chase & Myers, 2011) and more evenly distributed abundances among species (Passy, 2016). This would only have occurred in our experiment if the conditions on habitat islands deteriorated disproportionately among treatments, and specifically on all islands other than those that were large and near the adjacent forest. This can be refuted for the following reasons.

First, we surveyed habitat islands during the Austral spring (October; week 5), the height of summer (December and January; weeks 12 and 19), early autumn (April; week 24), and finally in summer of the following year (December; week 64). During this time, summer conditions were typical with temperatures routinely exceeding 35°C, whereas temperatures during spring and autumn rarely crept above 25°C. Rainfall was sporadic, less seasonal, and generally low over the course of the experiment with few rain days exceeding 10 ml. The most likely environmental stressor occurring in our experiment was therefore the more extreme temperature conditions during summer. This was unlikely to have contributed to the differences in abundance equality among habitat islands because it would have influenced conditions on all islands (as would any other seasonal stressor). Second, seasonal effects on animal communities were diminished inside the adjacent forest environment, but there was no indication that grassland communities near the forest edge experienced any comparable dampening of seasonal effects (Figure 4). Instead, our data were more likely the outcome of chance impacting dispersing individuals of some taxa to small and distant habitat islands. Determinism, on the other hand, had its most tangible effect at the level of species by influencing which taxa occurred on a particular sized habitat island, rather than generating within island differences in local abundance.

Conclusions on the relative contributions of deterministic and stochastic processes on the composition of species communities are contingent on resolving how those processes impact individual behavior and the distribution of species as a whole. Initially at least, the ecological requirements of species will determine the extent to which species will survive and reproduce in a new area. Adaptation might ultimately increase the “fit” of populations to their new environment (Blount, Borland, & Lenski, 2008; Lescak et al., 2015; Logan, Cox, & Calsbeek, 2014; Losos, Warheit, & Schoener, 1997), but if conditions differ enough from those experienced in the source environment, colonizers will fail to establish before adaptation has the opportunity to arise (Hayes & Barry, 2008; Hufbauer, Rutschmann, Serrate, Vermeil de Conchard, & Facon, 2013; Wolf, Garland, & Griffith, 1998). Ecologically similar residents can further restrict the establishment of invaders through competitive exclusion (Fargione, Brown, & Tilman, 2003; Fayle, Eggleton, Manica, Yusah, & Foster, 2015; Losos & Spiller, 1999; Schoener, 1983). However, the strength of environmental filters as a first order determinant of species distributions should be most apparent at large spatial scales where large environmental contrasts are most evident. The role of chance in colonization will also be evident at large spatial scales and is expected to result in the complete absence of poor dispersers from otherwise ecologically suitable habitat, and subsequently fewer numbers of species overall (Simberloff & Wilson, 1970; Crowell, 1973; Lomolino, 1982; Schoener & Schoener, 1983; reviewed by Warren et al., 2014). At small spatial scales, however, both environmental filtering and dispersal limitation are often assumed to be negligible because conditions are less likely to vary among adjacent habitats, and most species have a high probability of dispersing among nearby locations.

However, we were able to detect both deterministic and stochastic factors making separate contributions to the composition of animal communities over a small spatial scale (meters) and in a natural setting. Furthermore, had we not considered local abundances in our measures of diversity, we would have missed the signature of dispersal limitation in our data. Although determinism was clearly dominant in community formation, the ecological consequences of abundance inequality generated by dispersal limitation are not trivial. The number of individuals reaching a habitat (propagule size) and the size of the founded population are key predictors of colonization success (Lockwood, Cassey, & Blackburn, 2005; Simberloff, 2009) and the resilience of populations to local extinction (e.g. Schoener, Spiller, & Losos, 2001; Wootton & Pfister, 2013). The abundance of a subset of taxa can also have disproportionate flow‐on effects for the community as a whole. For example, the abundance of lower trophic levels affects the presence of higher trophic levels (e.g. predators can only follow the colonization of prey; Holt, 2009), and vice versa (Chase, Biro, Rybery, & Smith, 2009; Kneitel & Chase, 2004). Plasticity or generalist foraging behavior can reduce this dependency (Fahimipour & Anderson, 2015), but any limitation on the local abundance of certain taxa can profoundly affect the ecological resources available to other taxa (Harvey & MacDougall, 2014; Hein & Gillooly, 2011). What might seem like small effects of chance in the colonization history of one organism can have an extended effect on the abundance of, as well as the level of competition that might occur among, species within other trophic guilds (Chase, Burgett, & Biro, 2010; Fahimipour & Anderson, 2015).

Isolated habitats might ultimately reach their full ecological complement of species (carrying capacity) if poor dispersers have enough time to colonize those environments (Simberloff & Wilson, 1970). Temporal processes in community assembly are notoriously difficult to investigate without long‐term experimental study, which are rare (Table 1). On a basic level, deterministic factors could have lasting effects on community composition that outweigh those that initially occurred through dispersal limitation and chance (e.g. Hein & Gillooly, 2011; Li et al., 2016). In the future, we hope to continue monitoring the animal communities on our habitat islands to track the extent abundance inequalities among island diminish (or increase) with time, whether they are associated with increased species turnover over the long term (stochastic local extinction), and the extent to which local abundances and species diversity (or functional diversity; e.g. Magnago et al., 2014; Lefcheck & Duffy, 2015) are predictive of community resilience to experimental perturbations (e.g. denuding islands of all vegetation).

## AUTHOR C ONTRIBUTIONS

T. J. O. conceived and designed the experiment. T. J. O., M. H. and J. E. constructed habitat islands and transects. J. E. and T. J. O. collected the data. J. E., R. S., and S. S. sorted specimens. T. J. O. performed analyzes and wrote the paper. J. E., M. H., and R. S. provided editorial advice.

## CONFLICT OF INTEREST

None declared.
