# Supplementary material for: Manipulation of habitat isolation and area implicates deterministic factors and limited neutrality in community assembly
Source: Ecol Evol. 2017 Jun 20;7(15):5845–60. doi: 10.1002/ece3.3126 (PMC5552957; doi:10.1002/ece3.3126)
Supplement: Supplementary file 1 [file ECE3-7-5845-s001.docx]

**Supporting Information**

The following Supporting Information is available for this article online.

**Table S1.** Permutation ANOVAs of community dissimilarity of (a) small habitat islands and (b) large habitat islands to the surrounding grassland matrix (represented by the variable ‘habitat’, coded as 0 for matrix or 1 for islands). Variables with large statistically effects are highlighted in bold.

**Fig. S1.** Changes in vegetation cover from the initial construction of a habitat island to its final survey period for a representative (a) small and (b) large island. NB: both example islands were positioned near the forest edge (seen in the background of photos).

**Fig. S2.** Community dissimilarity between habitat islands and the surrounding grassland matrix for (a) the first 28 weeks and (b) final survey period at week 64.

**Table S1.**

| (a) Small islands vs transect stations | |  |  |  |  |
| --- | --- | --- | --- | --- | --- |
| All sampling methods, weeks 5 to 28* | | |  |  |  |
|  | Variable | d.f. | *F* | effect size (*r*) | *p* |
|  | **Week** | **1** | **5.97** | **0.29** | **0.001** |
|  | **Distance** | **1** | **1.55** | **0.15** | **0.05** |
|  | **Habitat** | **1** | **1.99** | **0.17** | **0.01** |
|  | week*distance | 1 | 1.11 | 0.13 | 0.31 |
|  | **week*habitat** | **1** | **2.17** | **0.18** | **0.01** |
|  | distance*habitat | 1 | 1.46 | 0.14 | 0.09 |
|  | week*distance*habitat | 1 | 0.92 | 0.11 | 0.55 |
|  | Residual | 55 |  |  |  |
|  | Total | 62 |  |  |  |
| Wet and dry pitfalls, week 64 | |  |  |  |  |
|  | Variable | d.f. | *F* | effect size (*r*) | *p* |
|  | habitat | 1 | 1.61 | 0.32 | 0.09 |
|  | distance | 1 | 0.88 | 0.24 | 0.54 |
|  | habitat*distance | 1 | 0.88 | 0.24 | 0.55 |
|  | Residual | 12 |  |  |  |
|  | Total | 15 |  |  |  |
| (b) Large islands vs transect stations | |  |  |  |  |
| All sampling methods, weeks 5 to 28* | | |  |  |  |
|  | Variable | d.f. | *F* | effect size (*r*) | *p* |
|  | **week** | **1** | **5.87** | **0.28** | **0.001** |
|  | **distance** | **1** | **1.90** | **0.16** | **0.02** |
|  | **habitat** | **1** | **4.07** | **0.24** | **0.001** |
|  | week*distance | 1 | 1.26 | 0.13 | 0.19 |
|  | **week*habitat** | **1** | **2.21** | **0.17** | **0.001** |
|  | distance*habitat | 1 | 1.17 | 0.13 | 0.29 |
|  | week*distance*habitat | 1 | 0.83 | 0.11 | 0.66 |
|  | Residual | 55 |  |  |  |
|  | Total | 62 |  |  |  |
| Wet and dry pitfalls, week 64 | |  |  |  |  |
|  | Variable | d.f. | *F* | effect size (*r*) | *p* |
|  | **habitat** | **1** | **2.41** | **0.37** | **0.02** |
|  | distance | 1 | 1.76 | 0.31 | 0.09 |
|  | habitat*distance | 1 | 1.70 | 0.31 | 0.09 |
|  | Residual | 12 |  |  |  |
|  | Total | 15 |  |  |  |

*One transect station at 50 m was excluded from the analysis because of persistent wombat disturbance (digging up of pitfalls and trampling of fly-paper glue-traps).

**Figure S1.**


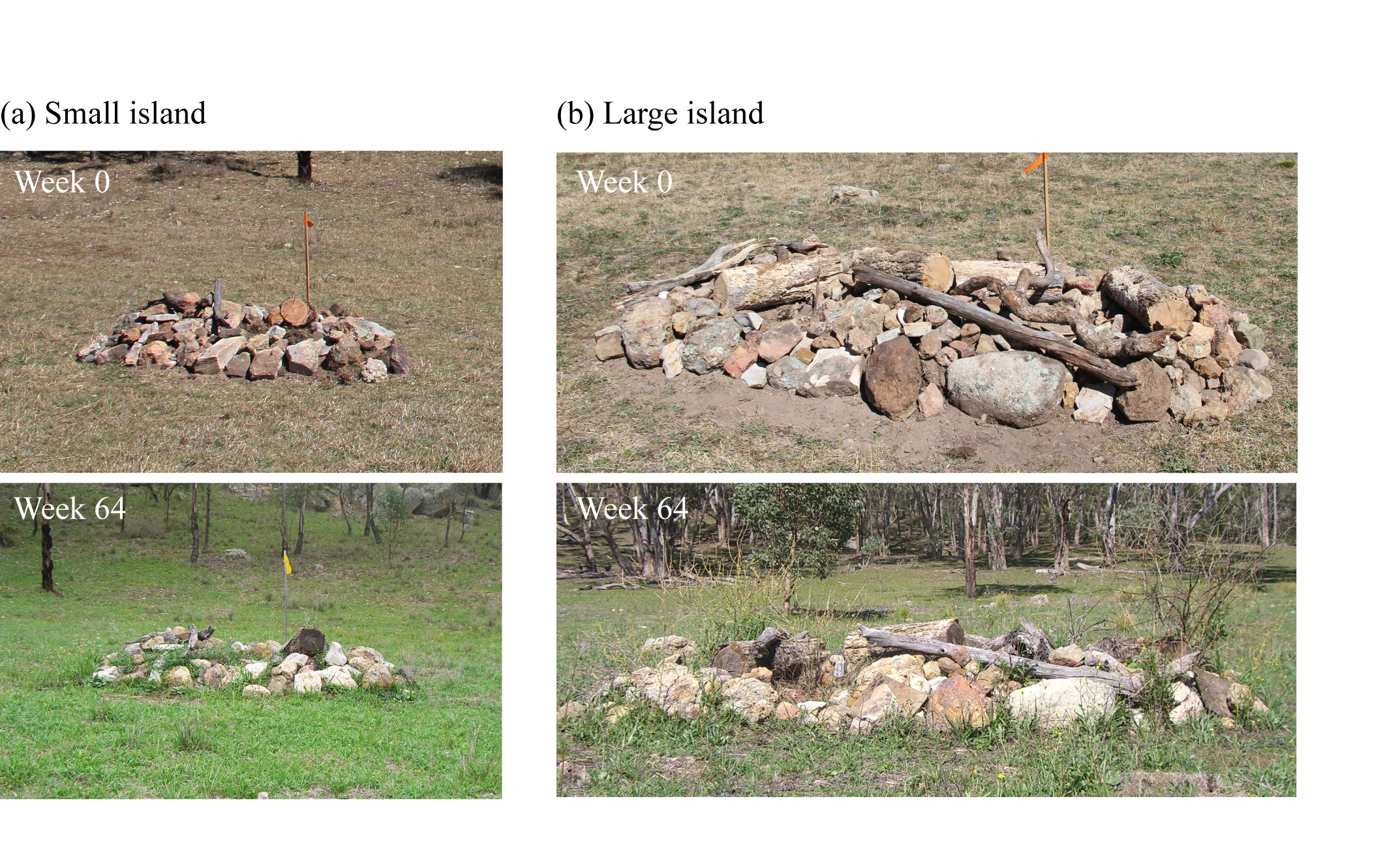


**Figure S2.**
